# Risk of post-intubation cardiac arrest with the use of high-dose rocuronium in COVID-19 patients with acute respiratory distress syndrome: A retrospective cohort study

**Published:** 2021-10-30

**Authors:** Natalie Kandinata, Roshan Acharya, Aakash Patel, Aalok Parekh, Jessica Santana, Aaron Darden, Yub Raj Sedhai, Smita Kafle, Usman Younus

**Affiliations:** ^1^Department of Internal Medicine, Cape Fear Valley Medical Center, Fayetteville, NC, 28306, United States of America; ^2^Department of Internal Medicine, Campbell University School of Osteopathic Medicine, Buies Creek, NC, 27506, United States of America; ^3^Department of Respiratory Therapy, Cape Fear Valley Medical Center, Fayetteville, NC, 28306, United States of America; ^4^Department of Internal Medicine, Virginia Commonwealth University School of Medicine, Richmond, VA, 23970, United States of America; ^5^Bachelor of Science in Nursing, Fayetteville State University School of Nursing, Fayetteville, NC, 28301, United States of America; ^6^Department of Critical Care Medicine, Cape Fear Valley Medical Center, Fayetteville, NC, 28306, United States of America

**Keywords:** COVID-19, acute respiratory distress syndrome, peri-intubation cardiac arrest, post-intubation cardiac arrest, rocuronium, rapid sequence intubation, etomidate

## Abstract

**Background:**

Post-intubation cardiac arrest (PICA) is an uncommon complication of intubation, but numbers have risen to over 1.5 times the usual number since the coronavirus disease 2019 (COVID-19) pandemic. Due to expert recommendations, high-dose rocuronium (HDR) has become a commonly used pre-intubation neuromuscular blocking agent.

**Aim:**

We conducted this retrospective case–control observational study with the hypothesis that high-dose rocuronium was not associated with higher incidence of PICA.

**Methods:**

We included 93 patients who were intubated using the rapid sequence intubation (RSI) technique with rocuronium for acute respiratory distress syndrome (ARDS) due to confirmed COVID-19 pneumonia, admitted from March 2020 to February 2021 to a tertiary care hospital in North Carolina, USA. The patients were grouped based on high (1.5 mg/kg of ideal body weight and above) versus low (<1.5 mg/kg of ideal body weight) dose rocuronium used for RSI. The differences of the various outcomes between the groups were analyzed.

**Results:**

The baseline demographics were similar in both groups except for higher body mass index in high-dose group 39 versus 32 (kg/m^2^), p = 0.009. There was a total of six PICA events (6.45%). The HDR group had 8.0% of PICA versus 4.7% in the low-dose group. In-hospital mortality was 60.0% in the HDR group versus 72.1% in the low-dose group.

**Conclusion:**

The incidence of PICA in COVID-19 patients with ARDS who were intubated using the RSI technique was higher than in the pre-COVID-19 era.

**Relevance for Patients:**

The use of high-dose paralytics during invasive ventilation with RSI and their consequences should be explored with the help of large-scale studies. The rate of PICA is still very low, and perhaps, the use of HDR is safe, as suggested by the expert panel.

## 1. Introduction

An early study from April 2020 reported that among 5700 patients hospitalized with coronavirus disease 2019 (COVID-19) in New York, 1151 (20%) required mechanical ventilation [1]. The INTUBE study, done before the COVID-19 pandemic, showed that 42.6% of intubated patients experienced peri-intubation cardiovascular instability, and 3.1% had post-intubation cardiac arrest (PICA). The primary reason for cardiac arrest in this study was hypovolemia or hemodynamic instability followed by hypoxemia. Following the start of the pandemic, PICA numbers have almost doubled, as seen in data derived from two large urban teaching hospitals [2].

At the beginning of the COVID-19 pandemic, in March 2020, experts in the field came together and published several pragmatic recommendations on managing critically ill patients with acute respiratory distress syndrome (ARDS) secondary to COVID-19 pneumonia. One of the general consensuses was to perform invasive mechanical ventilation with high-dose neuromuscular blocking agents such as 1.5 mg/kg of intravenous (IV) rocuronium or 2.0 mg/kg of IV succinylcholine with the goal of preventing incomplete or slow paralysis and minimizing the risk of high viral loads exposure found in sputum and upper airway secretions to the intubating provider [3,4]. The mechanism proposed behind this recommendation was to minimize cough reflex effectively through relief of laryngospasm to minimize virus transmission. There is a dose-dependent paralysis onset time for rocuronium, hence, 1.5 mg/kg of IV rocuronium was recommended [5]. Rocuronium has a longer “Safe Apnea Time” (SAT) compared to succinylcholine in obese patients [6]. Since patients who needed invasive mechanical ventilation were largely obese, rocuronium was preferred neuromuscular blocking in our center. However, the effect of high-dose rocuronium as a paralytic agent per recommendation on intubated COVID-19 patients has not been investigated.

This retrospective study aimed to look at the data objectively and determine if high-dose rocuronium was associated with higher rate of PICA. We hypothesized that high-dose rocuronium was not associated with higher PICA.

## 2. Materials and Methods

### 2.1. Study design and population

The study was a retrospective, case–control study that included patients admitted to three Cape Fear Valley Health System (CFVHS) hospitals from March 1, 2020, to February 28, 2021. The study was approved by the Institutional Review Boards (IRB, ID: NR1077-21) and was waived for the informed consent requirement. COVID-19 infection was confirmed by the reverse transcriptase-polymerase chain reaction (RT-PCR) test of the nasopharyngeal (NP) swab.

#### 2.1.1. Inclusion criteria

- Patients with severe acute respiratory syndrome coronavirus-2 (SARS-CoV-2) detected with RT-PCR method in the NP swab,
- Age 18 and above,
- Patients undergoing invasive mechanical ventilation using rapid sequence intubation (RSI) technique,
- Rocuronium used as neuromuscular blocking agent pre-intubation,
- Admission date between March 1, 2020, and February 28, 2021.

#### 2.1.2. Exclusion criteria

- SARS-CoV-2-negative RT-PCR test in the NP swab,
- Age <18 years,
- Pregnancy,
- The charts with incomplete information,
- Patients intubated post-cardiac arrest episode.

Patients were divided into high-dose rocuronium (HDR) group defined as 1.5 mg/kg and above, and low-dose rocuronium (LDR) group defined as doses below 1.5 mg/kg. The baseline demographics and comorbidities such as age, gender, race, body mass index (BMI), history of chronic obstructive pulmonary disease (COPD), cirrhosis, congestive heart failure (CHF), and diabetes mellitus (DM) were compared between the groups. Pre-intubation clinical characteristics such as acute physiology and chronic health evaluation (APACHE IV) score, pre-intubation ratio of arterial oxygen partial pressure to fractional inspired oxygen (PaO_2_:FiO_2_), oxygen saturation (SpO_2_), pre-intubation oxygenation method, respiratory rate, mean arterial pressure (MAP), heart rate (HR), shock index (SI), and number of vasopressors pre-intubation were also compared between groups. Basic laboratory markers included albumin, lactate dehydrogenase (LDH), ferritin, C-reactive protein (CRP), and D-dimer (DD).

The recommended standard dose of rocuronium for RSI was 0.6-1.2 mg/kg per manufacturer label (as approved by FDA). The standard high range of rocuronium was 1.2 mg/kg. The pragmatic recommendations were to use 1.5 mg/kg of rocuronium or 2 mg/kg of succinylcholine [4], which we opted for the study. The patients were intubated with etomidate as the anesthetic agent of choice. High versus normal dose etomidate was compared between the groups. The standard dose of etomidate was 0.3 mg/kg (per manufacturer box recommendation). High-dose etomidate (HDE) was defined as 0.4 mg/kg and higher. Our study used ideal body weight (IBW) for dosing purposes. The recorded time of rocuronium administration was considered 0 h (00:00) for PICA calculation. APACHE IV scores were obtained from the day of intubation.

### 2.2. Outcomes

#### 2.2.1. The primary outcome

PICA within 10 min of the intubation.

#### 2.2.2. The secondary outcomes

In-hospital mortality due to any cause; post-intubation PaO_2_:FiO_2_; post-intubation vasopressor requirement; mechanical ventilation duration; length of ICU stay; and length of hospital stay.

### 2.3. Data collection

The data were collected from the CFVHS electronic medical record system from March to April 2021 (2 months). The Microsoft Excel sheet was used to create the form for data collection. Three data miners were trained about the data mining methods, and two interim meetings were held to ensure the uniformity of data collection. The data were collected using the hospital computers and were stored in the health system’s secure internet network. The miners were not made aware of the allocation of the patient groups. The deidentified data were used for the statistical analysis.

### 2.4. Statistical analysis

Statistical analysis of continuous variables was reported as mean with standard deviation if normally distributed and as median with interquartile range if not normally distributed. Categorical variables were reported as frequency with percentage. Differences in continuous variables between the groups were evaluated with independent sample t-test or non-parametric Mann–Whitney U-test, as appropriate. Differences in the categorical variables between the groups were analyzed using Chi-square or Fisher’s exact test. All statistical tests of significance were two sided and conducted at the 0.05 level of significance. No sample size estimation was done as the study included all the samples that met the criteria for the defined time period. Statistical analysis was performed using STATA 16.1 (Stata Corp., College Station, TX).

## 3. Results

### 3.1. Demographic features and baseline characteristics

Ninety-three patients with confirmed respiratory failure secondary to ARDS due to COVID-19 pneumonia were intubated using RSI technique with rocuronium as the neuromuscular blocking agent was included in study. Fifty patients received high-dose rocuronium (≥1.5 mg/kg of IBW), and 43 patients received low dose (<1.5 mg/kg of IBW). The mean age of patients was 66 years, with 48% being female. Patients in the HDR group were more obese, with significantly higher BMI of 38.6 kg/m^2^ in the HDR group versus 32.7 kg/m^2^ in the LDR group (p = 0.009). The prevalence of common comorbidities such as COPD, cirrhosis, CHF, and DM was similar (Table 1). Albumin was lower in HDR at 2.2 versus 2.5 g/dL (p = 0.007), and inflammatory markers such as CRP (mean = 108 mg/L) were significantly elevated in both groups (Table 1). Patients in the HDR group received higher dose of etomidate more frequently, 87% versus 56% (p = 0.002).

**Table 1:** Baseline characteristic of patients in high- versus low-dose rocuronium groups

	All patients (n=93)	High-dose rocuronium (case group, n=50)	Low-dose rocuronium (control group, n=43)	p-value
Age (years), m±SD	66.11±11.51	64.64±11.82	67.84±11.01	0.183
Sex (female), n (%)	45 (48.39%)	30 (60.00%)	15 (34.88%)	0.016
BMI (kg/m^2^), m±SD	35.84±10.99	38.59±10.75	32.65±10.50	0.009
Race, n (%)				0.967
Caucasian	27.00 (29.03%)	14.00 (28.00%)	13.00 (30.23%)	
African-American	48.00 (51.61%)	26.00 (52.00%)	22.00 (51.16%)	
Other	18.00 (19.35%)	10.00 (20.00%)	8.00 (18.60%)	
Comorbid conditions, n (%)			
COPD	16.00 (17.39%)	8.00 (16.00%)	8.00 (19.05%)	0.701
Cirrhosis	3.00 (3.23%)	2.00 (4.00%)	1.00 (2.33%)	0.649
CHF	18.00 (19.35%)	8.00 (16.00%)	10.00 (23.26%)	0.377
Diabetes mellitus	51.00 (54.84%)	26.00 (52.00%)	25.00 (58.14%)	0.553
Albumin (g/dL), m±SD	2.33±0.46	2.20±0.46	2.46±0.44	0.007
LDH (units/L), m±SD	619.15±250.70	643.79±263.07	590.42±235.85	0.352
Ferritin (ng/mL), m±SD	1031.88±1101.18	1079.26±1350.24	972.66±687.90	0.668
CRP (mg/L), m±SD	107.94±61.51	119.52±59.67	93.48±61.51	0.058
D-Dimer (mcg/mL), m±SD	6.36±9.79	5.90±7.65	6.91±11.99	0.647
APACHE IV, m±SD	61.53±21.05	62.10±21.60	60.95±20.76	0.811
Pre-intubation SpO_2_ (%), m±SD	91.20±7.73	90.79±7.67	91.65±7.87	0.603
Pre-intubation PaO_2_:FiO_2_ (mmHg), m±SD	90.26±62.25	100.03±76.83	78.39±35.19	0.118
Pre-intubation RR, m±SD	34.13±11.40	35.68±13.36	32.33±8.38	0.158
Pre-intubation HR, m±SD	105.19±23.53	107.34±23.85	102.70±23.18	0.346
Pre-intubation MAP, m±SD	101.44±21.71	100.33±20.96	102.73±22.72	0.597
Pre-intubation SI, m±SD	0.77±0.29	0.80±0.35	0.73±0.19	0.212
Pre-intubation oxygenation method with NIV, n (%)	81.00 (87.10%)	42.00 (84.00%)	39.00 (90.70%)	0.337
Pre-intubation vasopressors requirement, n (%)				0.603
0 vasopressor	80.00 (86.02%)	42.00 (84.00%)	38.00 (88.37%)	
1 vasopressor	12.00 (12.90%)	7.00 (14.00%)	5.00 (11.63%)	
2 vasopressors	1.00 (1.08%)	1.00 (2.00%)	0.00 (0.00%)	
HDE (>0.3 mg/kg), m±SD	54.00 (72.00%)	34.00 (87.18%)	20.00 (55.56%)	0.002
Rocuronium dose (mg/kg), m±SD	1.50±0.53	1.85±0.41	1.10±0.33	<0.001

n: Number; m: Mean; SD: Standard deviation; BMI: Body mass index; COPD: Chronic obstructive pulmonary disease; CHF: Congestive heart failure; LDH: Lactate dehydrogenase; CRP: C-reactive protein; APACHE IV: Acute physiology and chronic health evaluation; SpO_2_: Oxygen saturation (%); PaO_2_:FiO_2_: Ratio of arterial oxygen partial pressure (mmHg) to fractional inspired oxygen; RR: Respiratory rate (breaths per minute); HR: Heart rate (beats per minute); MAP: Mean arterial pressure (mmHg); SI: Shock index; NIV: Non-invasive ventilation; HDE: High-dose etomidate

### 3.2. Primary outcome

Six percent (6/93) of patients intubated with rocuronium had PICA. Four patients in high dose versus two patients in low dose (8% vs. 4%) were found to have PICA.

### 3.3. Secondary outcomes

Sixty-six percent (61/93) of those with COVID-19 respiratory failure who were intubated with rocuronium as part of the RSI regimen had in-hospital mortality. The incidence of in-hospital mortality was less in patients who received high-dose rocuronium 60% versus 72%, though the comparison was not clinically significant. There was no difference in post-intubation PaO_2_:FiO_2_, post-intubation vasopressors requirement, mechanical ventilation duration, length of ICU stay, and length of hospital length between both groups (Table 2).

**Table 2:** Post-intubation clinical outcomes in high- versus low-dose rocuronium groups

	All patients (n=93)	High-dose rocuronium (case group, n=50)	Low-dose rocuronium (control group, n=43)	p-value
PICA within 10 min, n (%)	6.00 (6.45%)	4.00 (8.00%)	2.00 (4.65%)	0.512
In-hospital mortality, n (%)	61.00 (65.59%)	30.00 (60.00%)	31.00 (72.09%)	0.221
Post-intubation PaO_2_:FiO_2_ (mmHg), m±SD	110.40±68.78	109.42±72.57	111.55±64.91	0.884
Post-intubation vasopressor requirement, n (%)				0.452
0 vasopressor	38.00 (41.30%)	21.00 (42.86%)	17.00 (39.53%)	
1 vasopressor	44.00 (47.83%)	23.00 (46.94%)	21.00 (48.84%)	
2 vasopressors	8.00 (8.70%)	5.00 (10.20%)	3.00 (6.98%)	
3 vasopressors	2.00 (2.17%)	0.00 (0.00%)	2.00 (4.65%)	
Ventilator hours, m±SD	275.14±282.06	269.87±311.32	281.14±248.08	0.850
Ventilator days, m±SD	11.46±11.75	11.24±12.97	11.71±10.34	0.849
ICU LOS, m±SD	12.49±12.76	12.21±14.40	12.81±10.72	0.822
Hospital LOS, m±SD	20.91±16.76	19.99±18.10	22.00±15.18	0.565

n: Number; m: Mean; SD: Standard deviations; PICA: Post-intubation cardiac arrest; PaO_2_:FiO_2_: Ratio of arterial oxygen partial pressure (mmHg) to fractional inspired oxygen; LOS: Length of stay in days

## 4. Discussion

In this single health system study, we found that the PICA incidence had increased to almost double compared to the pre-COVID-19 era. There was also no difference in in-hospital mortality with the use of the high dose of rocuronium during RSI.

The data related to incidence and circumstances leading to PICA in patients with COVID-19 pneumonia are limited. Due to the novelty of the virus, the recommendation was based on expert opinion rather than evidence based. Some of these recommendations included giving high-dose paralytic and early intubation using the RSI technique, if the defined parameters were met [4]. The reasoning behind the recommendation to use higher dose of rocuronium was because of the dose-dependent onset of effect of rocuronium during RSI. The standard dose of rocuronium (0.9-1.2 mg/kg) was recommended for optimal intubation conditions within 60 seconds of neuromuscular blocking agent administration [7]. A prospective study on patients with burn injuries which lead to resistance to the effects of non-depolarizing muscle relaxants showed that 1.5 mg/kg rocuronium dose produced initial onset of paralysis as early as 30 seconds, which can be speculated to be a reasonable onset of time for relief of laryngospasm [5]. Theoretically, less laryngospasm minimizes the aerosol transmission of the virus to the intubating provider, hence, maximum relief is favored at minimum time. The effect of the high dose of rocuronium during RSI has not yet been studied in patients with ARDS secondary to COVID-19 pneumonia. However, there has been a reported significant increase in the incidence of PICA by almost double since the pandemic started. Our study was consistent with this finding as compared to INTUBE study (6.4 vs. 3.1%) [2].

Interestingly, there has been a trend toward higher than standard dose etomidate (>0.3 mg/kg of IBW) usage during RSI in our health system. Etomidate has an effect of mild hypotension and agonistic action on gamma aminobutyric acid (GABA) receptors that can result in adrenal insufficiency [8]. This high dose of etomidate possibly might have contributed to immediate worse outcomes such as hemodynamic instability or PICA in the patients who needed mechanical in ARDS due to COVID-19 pneumonia, but so far, there have been no studies to support this theory.

Obesity is well known to be associated with reduced lung function and poor response to mechanical ventilation, higher inflammatory states in the body, and overall, more severe symptoms and poor prognosis in COVID-19 patients [9]. Although the number of first time intubation failures in general did not differ in patients having mechanical intubation with rocuronium versus succinylcholine before the COVID-19 pandemic, rocuronium has the advantage of having longer SAT in overweight and obese patients [6,10]. SAT is defined as the amount of apnea time allowed before a patient desaturates below 92% or reaches a blood oxygen saturation level of 88-90% [6,11]. Since most patients who needed mechanical ventilation were obese, rocuronium was preferred over succinylcholine for performing mechanical intubation in our health system as it allowed longer SAT. Prolonging the apneic window without desaturating oxygen level allows providers to change the nature of their airway management, in the event unanticipated complications arise [12].

Another factor to consider regarding the higher incidence of PICA during this COVID-19 pandemic could be hypercapnia during SAT. It is important to realize that in addition to low oxygenation levels, severe acidosis caused by increased PCO_2_ can occur during the apnea time, leading to cardiac arrest if it could not be addressed in time [13,14]. Therefore, it would be interesting to see studies on ideal initial tidal volume during mechanical ventilation to correct hypercapnia accumulated during SAT in COVID-19 patients and its effect on PICA. A study suggests the impact of acute hemodynamic instability from autonomic imbalance triggered by the procedures and medications administered during the procedure in critical care settings on mortality [15]. There remains the possibility that similar mechanisms could have contributed to PICA in certain patients.

There were a few limitations to our study. First, the sample size was small, given that it was a single health system study. However, since the primary outcome was an uncommon endpoint, we anticipated a low sample. Second, due to only six PICA events, we could not perform multivariable logistic analysis. We could not access the association of hypoalbuminemia, obesity, and elevated inflammatory markers, which have been associated with worse adverse outcomes in various diseases and COVID-19 infection, with the PICA outcome [16-23]. Third, we could not divide patients into mild, moderate, and severe ARDS and analyze the association on PICA outcome due to only six PICA events. However, this is the first study of its kind to compare high versus low rocuronium doses during RSI in COVID-19 pneumonia patients who required mechanical ventilation due to ARDS. The use of HDR in this setting seems to be justified as it minimizes the exposure risks to providers.

## 5. Conclusion

In comparison to the pre-COVID-19 era, the incidence of PICA had increased among COVID-19 patients with ARDS who were intubated with high-dose rocuronium using RSI technique. Further study to explore the association of high-dose paralytic agents with PICA needs to be done.

### Conflicts of Interest

The authors declare that there are no conflicts of interest.
